# Comment on "Overweight status, abdominal circumference, physical activity, and functional constipation in children"

**DOI:** 10.1590/1806-9282.20240603

**Published:** 2024-09-02

**Authors:** Baogui Wang, Haibo Xu

**Affiliations:** 1 Shandong Women's University – Jinan, China.

Dear Editor,

We would like to share a few thoughts on the study titled "Overweight status, abdominal circumference, physical activity, and functional constipation in children."^1^ The objective of this study was to assess the prevalence of functional constipation and its association with food intake, overweight status, and physical activity in children. In this study, 452 children aged 6–12 years from two public schools were evaluated using a cross-sectional study. First, functional constipation was diagnosed based on Rome IV criteria clinical presentation for more than 2 months. Next, food intake, body mass index, height-for-age z-score, abdominal circumference, abdominal circumference for height, and physical activity indicators were tested separately. The results showed that a larger abdominal circumference was associated with functional constipation in girls (p=0.036) and boys induced by increase in consumption of fat (p=0.041). However, there are two issues that require further elaboration.

First, we believe that the overall study design is flawed. However, the choice of a cross-sectional study for this study is not appropriate with the study content, after all, the occurrence of functional constipation is a long period of time, and it is not reasonable to just intercept the data for a certain period of time. For example, in the food intake analysis, the study used a 24-h dietary recall survey. We believe that obtaining data in this way is incomplete, and simply investigating dietary status over a 24-h period can easily lead to inaccurate results. Therefore, we suggest the authors repeat the use of 24-h dietary recall survey to collect food intake data for analysis in the established research cycle to ensure the randomness and accuracy of the data.

Second, the study's finding that "functional constipation is associated with a larger abdominal circumference in girls" is challenged. The study ignored the fact that the amount of abdominal fat is a key factor in abdominal circumference^2^ and thus did not specifically address the question of the amount of abdominal fat in the respondents. If the girls in the study had increased abdominal circumference due to the accumulation of subcutaneous fat, the results of this study would have resulted in false positives. Therefore, we recommend adding the measurement of abdominal fat content to rule out such false positive results.
